# Molecular docking and dynamic simulations of benzimidazoles with beta-tubulins

**DOI:** 10.6026/97320630017404

**Published:** 2021-03-31

**Authors:** Chennu Maruthi Malya Prasada Rao, Narapusetty Naidu, Jhansi Priya, K Poorna Chandra Rao, Kapu Ranjith, Singarapalle Shobha, Bodepudi Sudheer Chowdary, Sridhar Siddiraju, Sabitha yadam

**Affiliations:** 1 Department of Pharma Chemistry, QIS College of Pharmacy, Ongole, AP, India-523272; 2 Department of Pharma Chemistry Bellamkonda Institute of Technology and Science, Podili, AP, India-523240; 3 Department of Pharma Chemistry Baptla College of Pharmacy, Bapatla, AP, India-522201; 4 Department of Pharma Chemistry Malla Reddy College of Pharmacy, Secunderabad, Telangana India-500014; 5 CEO and Founder, Ciencia Life Sciences, Kukatpally, Hyderabad, India

**Keywords:** Benzimidazole, beta-tubulins inhibitors, anthelmintic activity, albendazole, colchicine domain, microtubule

## Abstract

It is of interest to document the molecular docking and dynamic simulations of benzimidazoles with beta-tubulins in the context of anthelmintic activity. We document the compound BI-02 (2-(3,4-dimethyl phenyl)-1H-1,3-benzimidazole (BI-02) with optimal bindig features compared to the standard molecule albendazole (7.0 Kcal/mol) with binding energy -8.50 Kcal/mol and _P_IC_50_ value 583.62 nM.

## Background

Microtubules (MT) are tubular structured protein polymers that form part of the cytoskeleton within cells composed of subunits of the protein tubulin. They are associated with the mitotic spindle, centrioles, neurotubules, cilia, and flagella and are necessary to maintain cell shape and transport material within cells and mitosis [1]. Tubulin has been isolated from all vertebrates and many invertebrates, e.g., fungi, helminths, and plants. The tubulin molecules isolated from these diverse sources are closely related but not identical. The microtubule cytoskeleton participates in almost all cellular processes, including cell motility, cargo transport, cell division and morphology maintenance or changes [2]. Both α and β tubulin can bind GTP, α tubulin lacks appreciable hydrolysis activity, and nucleotide turnover is slow in comparison, nucleotide exchange in β tubulin is fast at physiological Mg2+ concentrations [3]. Tamm, Folkers, and co-workers in 1954, first synthesized halogenated benzimidazole nucleosides as antiviral compounds [4]. Since the 1960s, Benzimidazoles have been used as anthelmintic agents in agriculture as antifungal agents and in human medicine and veterinary medicine [5]. Benzofuran derivatives of benzimidazole moiety were available naturally, and synthetic bioactive compounds such as pesticide insecticide, in vitro cytotoxic, anti-inflammatory, antimicrobial antioxidant, anti-HIV-1 and anticancer agents [6, 7]. The benzimidazole ring system is a widely used pharmacophore in medicinal chemistry and current drug discovery. Albendazole [8] and thiabendazole [9] are anthelmintic drugs that act by the inhibition of tubulin polymerization and impair glucose uptake, eventually leading to the death of the parasites. The benzimidazole structures were chosen because they had a broad spectrum of anthelmintic activity [10,11]. Prichard et al. [12] determine the molecular docking activity aquarium with Tubifex tubifex against the tubulin-colchicine enzymes having a concentration the 11.90 mg/mL. Yadav D. Bodke et al. [6] performed in vivo evaluation of thiazole-benzimidazole nucleus contains benzofuran derivatives shows potential anti-fungal and anti-helminthic activity. Salgado et al. used chemoinformatic tools to investigated benzimidazole-derived drugs as potential treatments for leishmaniasis. [13] Therefore, it is of interest to document the molecular docking and dynamic simulations of benzimidazoles with beta-tubulins in the context of anthelmintic activity in comparison to the standard compound Piperazine citrate [14].

## Methodology

### Software and programs:

Accelrys Discovery studio version 4.0 [15-16] is utilized to visualize the ligand structures, receptors, and hydrogen-bonding networks. It is also used to render images. Six benzimidazoles ligand structures were drawn using the Chemsktech software [17] and were converted to 3d format saved to mol2 format for further processing. All ligands were Energy minimized by chimera [18] applying 'AMBER' force field with steepest descent algorithm. Protein was collected from RCSB bank (www.rcsb.org) in PDB ID: 1SA0 (Crystal structures of tubulin complexed with colchicine binding site selected for this study) [19].

### OSIRIS Property Explorer:

OSIRIS Property Explorer [20-21] was used to estimate Drug-likeness and toxicity predictions, risks of side effects, such as tumorigenic, mutagenic, irritant and reproductive effects, as well as drug-relevant properties like LogS (solubility), MW (Molecular weight), cLogP, and overall drug-score (REF). Overall drug-score was calculated by combining cLogP, LogS, MW, toxicity risks, and drug-likeness outcome.

### Docking:

Autodock 4.0 [22] is the primary docking program used for semi-flexible docking studies. Preparation of the ligands and protein receptors in pdbqt file and determination of the grid box size was carried out using Autodock Tools version 1.5.6. A grid box of size 90Åx90Åx90Å with a spacing of XYZ grid center 119.684 90.098 5.767 XYZ coordinator are auto at the αβ tubulin interface, i.e., the putative colchicine binding site the protocol used for performing Protein and ligand preparation, along with docking studies, are described elsewhere [23].

### Molecular simulations: 

Schrodinger's Desmond module Ver 3.6 was utilized to perform the classic MD simulations and it's analysis [24,25].

### Docking complex simulation:

The molecular dynamic simulations were carried out for docked protein ad ligand complexes to study the stability of benzimidazoles ligand with tubulin a chain (binding site of colchicine). All simulations were performed using Desmond v3.6 Package [25].

### Pre-processing and preparation of protein target structure and ligand:

The Crystal Structure of the human Tubulin complex with colchicine Protein [1SA0] was resolved using X-ray diffraction method with a resolution factor of 3.58 Å was retrieved from PDB Retrieved structure, which has been further modified for docking calculations, and Protein was imported to Maestro v9.6 (Maestro, version 9.6, Schrodinger, LLC, New York, NY, 2013) [2]. Using Protein Preparation Wizard (PPW) included biological units and assigned bond orders, created disulfide bonds, deleted all water molecules, generated metal-binding states for heteroatoms, added missing hydrogens. We didn't find any breaks and missing atoms in the protein crystal structure. Preparation of the ligands for docking studies using LigPrep Ver 2.8 module LigPrep, version2.8 (Schrodinger, LLC, New York, NY, 2013) the Schrodinger suite 2013.3. The pH range was set to 7.0, and applying the OPLS2005 force field minimized the Protein and ligand.

## Results and Discussion:

**Figure 1:**
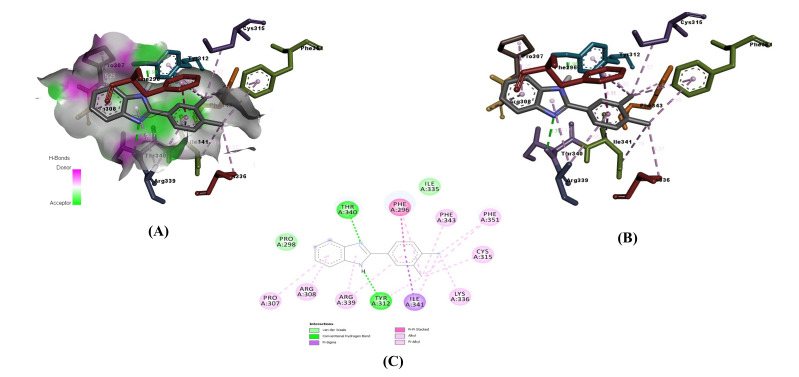
Docking snapshots of the best binding compound BI-02 with β-Tubulin. (A) 3D-Image of BI-02 with the protein and (B) Molecular interactions of BI-02 with protein C) 2-D Image BI-02 interactions of with the protein.

**Figure 2:**
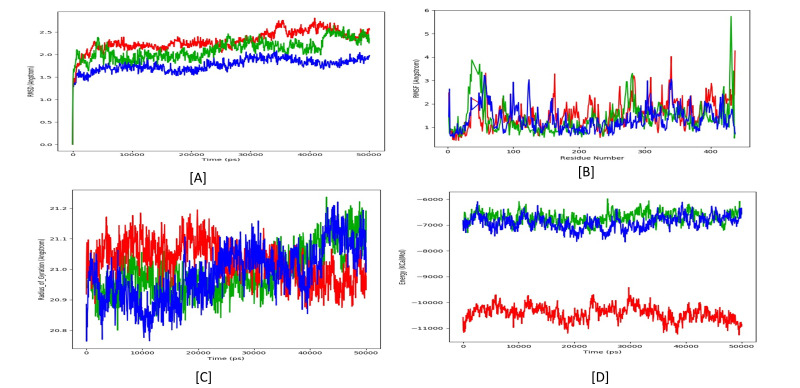
[A] RMSD graph of Tubulin in it's apo form (red), in complex with Colchicine (Green), and in complex with BI-02 (Blue), [B] RMSF graph of Tubulin in it's apo form (red), in complex with Colchicine (Green) and in complex with BI-02 (Blue), [C] Radius of Gyration graphs of Tubulin in it's apo form (red), in complex with Colchicine (Green) and in complex with BI-02 (Blue) [D] Energy graph in Kcal/mol of Tubulin in its apo form (red), in complex with Colchicine (Green) and in complex with BI-02 (Blue).

**Figure 3:**
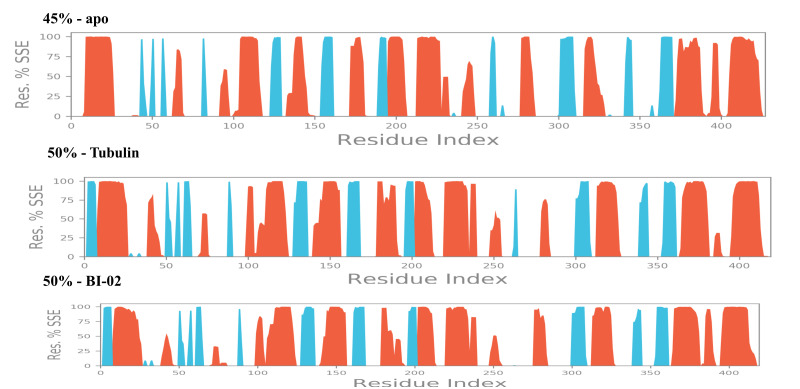
RMSF graph of tubulin in it's apo form (top), in complex with Colchicine (middle) and in complex with BI-02 (bottom).

**Figure 4:**
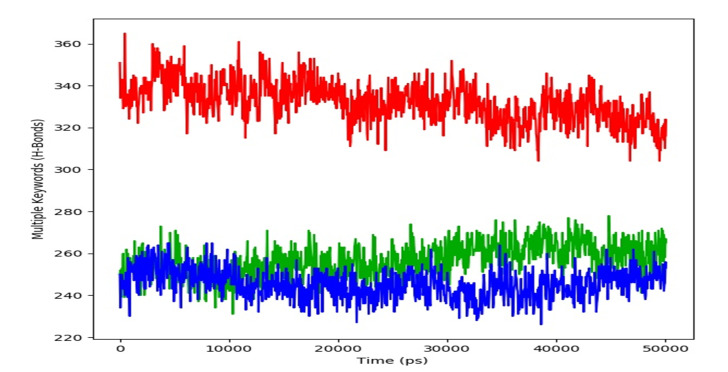
Total number of intramolecular hydrogen bonds formed within the Tubulin in it's apo form (red), in complex with Colchicine (Green) and in complex with BI-02 (Blue).

**Figure 5:**
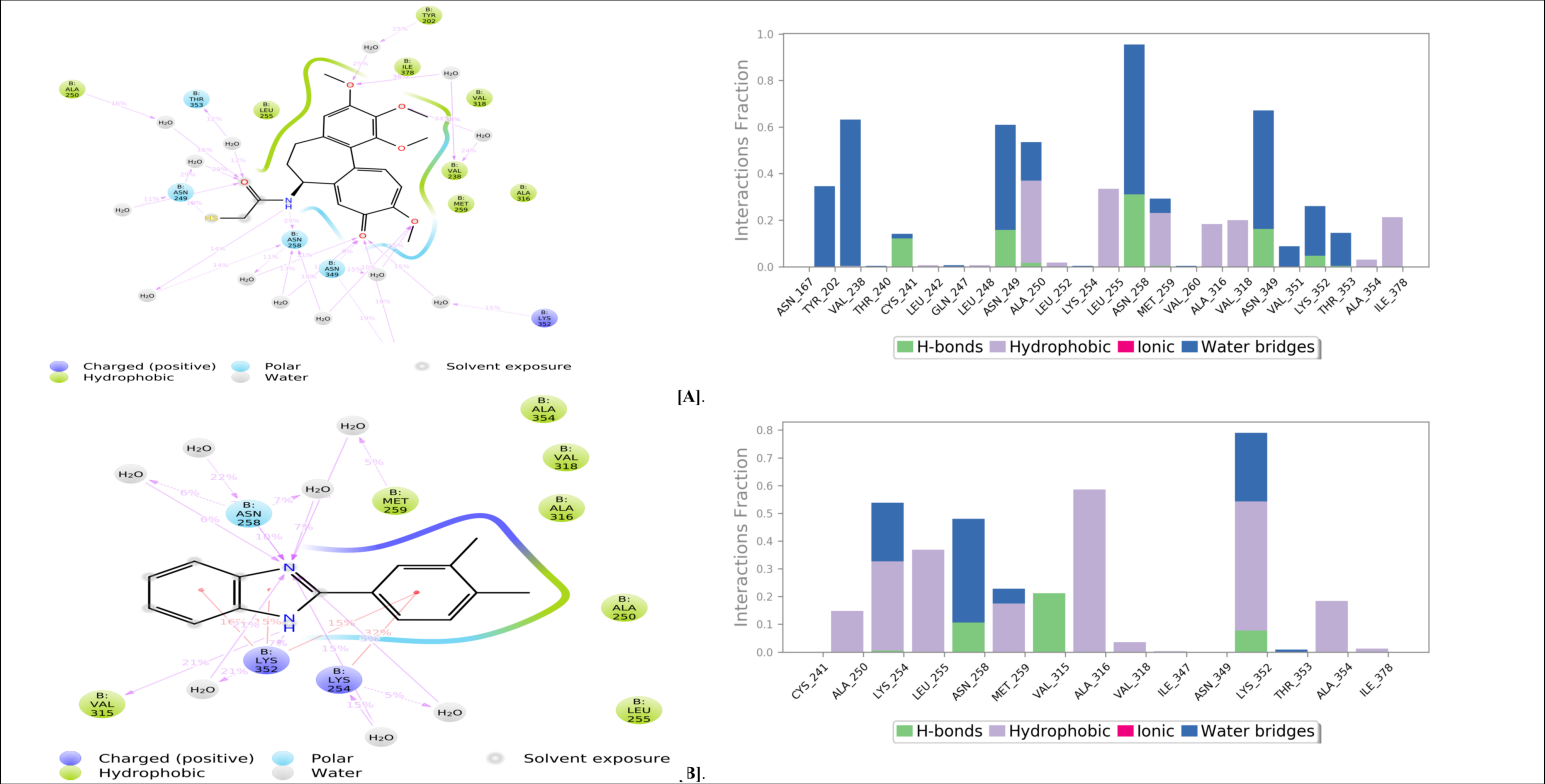
(A) Molecular interactions observed between tubulin and Colchicine during the simulation, (B) Molecular interactions observed between tubulin and BI-02 compound during the simulation.

In this study, beta-tubulin protein and six selected benzimidazoles molecular docking simulation was investigated and analysed the effective docked ligand. All the six compounds show successful docking inside the active site of the beta-tubulin protein (1SA0) with binding energies ranging from -7.11 Kcal/mol to -8.50 Kcal/mol and with predicted inhibitory concertation of ranging from 1.40 uM BI-02 interacted with amino acid of active sites of beta-tubulin by two hydrogen bonds with THR A: 340, TYR A: 312, Pi-Pi interactions with PHE A:296, Pi-Sigma bond with ILE A: 341, the Pi-Alkyl interactions with the PHE A:343, PHE A:351, CYS A:315, ARG A: 308, ARG A: 339, Alkyl interaction LYS A: 336, Vander wall interaction with PRO A:298 respectively with the Protein and interactions were shown in Figure 1. The molecular descriptors values reveal that the compound BI-02 obeys the Lipinski rule of five and Veber rule of five and no toxicity. The results were tabulated in Table 2,Table 3. In the present work, OSIRIS Property Explorer open-source program [14] was used to predict risks of side effects, such as mutagenic, tumorigenic, irritant and reproductive effects of selected six compounds. Interestingly, the potential drug-likeness values of all compounds were significant and nontoxic (Table 2 and Table 3). MD simulations were executed to confirm the binding energy and molecular level interactions determine in the molecular docking. Initially, we performed individual MD simulations for apo form, beta-tubulin complexed with colchicin and with the best compound identified in this study i.e., BI-02 (2-(3,4-dimethyl phenyl)-1H-1, 3-benzimidazole). The Root mean square deviation (RMSD) of proteins backbone was observed to be fluctuating between 1.5 and 2.5 Å (Figure 2A) throughout the simulated timescale of 50ns each. Among the three simulations, the apo form of the Protein was found to be fluctuating the highest up to 2.5 Å, whereas the Protein in complex with Colchicine was found to be maintaining an average of 2.0 Å, clearly showing that the Protein is much stabilized in its activity in the presence of the Colchicine. Nevertheless, the best stability of the Protein was observed in sight of the BI-02 compound with an average of 1.7 Å. Based on n the RMSD analysis, Root mean square fluctuations (RMSF) of the Protein individual residue (Figure 2B) were also found to be co-ordination. The Protein residual level movements were minimized in their movement/fluctuations in Protein in complex with BI-02 compound compared to its apo form and in complex with Colchicine. The Gyration radius is another measure we took note of for the Protein's simulated time scale in the above mentioned three cases and observed that the Protein maintains an average of 21.1, 21.0, and 20.9 angstroms, with few significant fluctuations observed, as shown in Figure 2C. The much-minimized RMSD and RMSF indicate that the BI-02 compound has a better inhibitory influence on the protein activity. The same can be seen affirmed by the minimized energies observed throughout the simulated timescale from -11000 Kcal/mol in its apo form protein energy to around an average of -7000 and -6800 Kcal/mol of Energy for Protein in complex with Colchicine and in complex with BI-02 respectively (Figure 2D). We have also monitored the total secondary structure elements (SSE) like alpha helices and beta strands present in the Protein throughout the simulation trajectory. The analysis revealed that the Protein in the complex with Colchicine and BI-02 compound was maintaining an average of 50% of SSE composition (Figure 3) compared to 45% of SSE composition in its apo form made of helices and strands throughout the simulated time. Most of the Protein is stabilized with strands (blue), helices (red), and loops (white). We have monitored the total number of intramolecular hydrogen bonds formed within the Protein in its apo form compared to Protein in complex with Colchicine and in complex with BI-02 and observed that the Protein was maintaining an average of 330, 260 and 240 respectively (Figure 4). 24 intermolecular contacts between tubulin and colchicine during the simulated time scale is seen. Among which, 10 contacts were involved in hydrophobic interactions, 12 contacts in polar interactions and about eight direct hydrogen bonds. The residues involved in hydrophobic contacts are CYS241, LEU248, ALA250, LEU252, LEU255, MET259, ALA316, VAL318, ALA354, and ILE378. The residues found to form polar interactions mediated by water molecules are AS167, Tyr202, Cys241, Gln247, Asn249, Ala250, Asn258, Met259, Asn349, Val351, Lys352 and Thr353. And finally, the residues involved in direct hydrogen bonding are Thr240, Cys241, Asn249, Ala250, Asn258, Val260, Asn349 and Lys352 (Figure 5A). 15 intermolecular contacts between tubulin and BI-02 compound during the simulated time scale. Among these, 11 contacts were involved in hydrophobic interactions, 5 contacts in polar interactions, and four direct hydrogen bonds. The residues involved in hydrophobic contacts are Cys241, Ala250, Lys252, Met259, Ala316, Val318, Ile347, Asn349, Lys352, Ala354, and Ile378. The residues found to form polar interactions mediated by water molecules are Lys254, Asn258, Met259, Lys352, and Thr353. And finally, the residues involved in direct hydrogen bonding are Asn258, Val315, and Lys352 (Figure 5B). To759.23 nM concentrations, and compared with the standard compound albendazole value of −7.0 Kcal/mol. The compound BI-02 shows better activity among the six compounds with -8.50 Kcal/mol binding energy and PIC50 value 583.62 nM (Table 1).

## Conclusion

We document the compound BI-02 (2-(3,4-dimethyl phenyl)-1H-1,3-benzimidazole (BI-02) with optimal bindig features compared to the standard molecule albendazole (7.0 Kcal/mol) with binding energy -8.50 Kcal/mol and PIC50 value 583.62 nM for further consideration in this context.

## Figures and Tables

**Table 1:** Docking results of the compounds BI_01 to BI_06 with Beta-Tubulin (PDB ID: 1SA0)

S.No	Compound	Docking energy (Kcal/mol)	PIC50 value
1	2-phenyl-1H-benzimidazole (BI-0)	-7.39	3.84 uM
2	2-(3,4-dimethylphenyl)-1H-benzimidazole (BI-02)	-8.5	583.62 nM
3	2-(4-chloro-3-nitrophenyl)-1H-benzimidazole (BI-03)	-8.35	759.23 nM
4	4-(1H-benzimidazol-2-yl)aniline (BI-04)	-7.11	6.18 uM
5	2-(4-nitrophenyl)-1H-benzimidazole (BI-05)	-7.76	2.04 uM
6	2-(4-chlorophenyl)-1H-benzimidazole (BI-06)	-7.99	1.40 uM

**Table 2:** The molecular descriptor values of the compounds.

S.No	Compound	Molecular Formula	Mol. Wt.	Log P	No. of H-bond donors	No. of H-bond acceptors	No. of rotatable bonds	TPSA	ADME Pass / Fail
1	BI-01	C13H10N2	194.236	2.9022	1	2	1	28.68	PASS
2	BI-02	C15H14N2	222.29	3.59	1	2	1	28.68	PASS
3	BI-03	C13H8N0O2Cl	273.679	2.5866	1	5	2	74.5	PASS
4	BI-04	C13H11N3	209.251	2.2249	2	3	1	54.7	PASS
5	BI-05	C13H9N3O2	239.233	1.9806	1	5	2	74.5	PASS
6	BI-06	C13H9N2Cl	228.681	3.5082	1	2	1	28.68	PASS

**Table 3:** Toxicity of compounds based on Osiris Property Explorer predictions.

S.No	Compound	Mutagenic	Tumorigenic	Reproductive effective	Irritant
1	BI-01	None	None	None	None
2	BI-02	None	None	None	None
3	BI-03	None	None	None	None
4	BI-04	None	None	None	None
5	BI-05	None	None	None	None
6	BI-06	None	None	None	None
